# Prognostic analysis of MDA5‐associated clinically amyopathic dermatomyositis with interstitial lung disease

**DOI:** 10.1002/iid3.1332

**Published:** 2024-06-27

**Authors:** Wen Wang, Xiang Sun, Yan Xu, Wenfeng Tan, Ye Liu, Jun Zhou

**Affiliations:** ^1^ Department of Rheumatology and Immunology The Affiliated Suqian First People's Hospital of Nanjing Medical University Suqian China; ^2^ Expanded Program on Immunization Jiangsu Provincial Center for Disease Control and Prevention Nanjing China; ^3^ Department of Rheumatology The First Affiliated Hospital of Nanjing Medical University Nanjing China; ^4^ Department of Pharmacy The Affiliated Suqian First People's Hospital of Nanjing Medical University Suqian China

**Keywords:** anti–melanoma differentiation–associated gene 5, clinically amyopathic dermatomyositis, interstitial lung disease, prognosis

## Abstract

**Objective:**

To investigate the prognostic factors of patients with anti‐melanoma differentiation‐associated gene 5 (MDA5) positive clinically amyopathic dermatomyositis (CADM) and interstitial lung disease (ILD).

**Methods:**

A retrospective analysis was conducted on clinical data of 125 patients with anti‐MDA5 + CADM‐ILD collected from 10 branches in eastern China between December 2014 and December 2022. Prognostic factors were analyzed using *χ* ^*2*^ test, Log‐rank test, COX and logistic regression analysis.

**Results:**

In this cohort, 125 anti‐MDA5 + CADM‐ILD patients exhibited a rapidly progressive interstitial lung disease (RPILD) incidence of 37.6%, and an overall mortality rate of 24.8%. One patient was lost to follow‐up. After diagnosis of RPILD, a mortality rate of 53.2% occurred in patients died within 3 months, and that of 5.6% appeared in those who survived for more than 3 months. Multiple factor analysis revealed that C‐reactive protein (CRP) ≥ 10 mg/L (*p* = 0.01) and recombinant human tripartite motif containing 21 (Ro52) (+) (*p* = 0.003) were associated with a higher risk of RPILD in anti‐MDA5 + CADM‐ILD patients; CRP ≥ 10 mg/L (*p* = 0.018) and the presence of RPILD (*p* = 0.003) were identified as the factors influencing survival time in these patients, while arthritis was the protective factor (*p* = 0.016).

**Conclusion:**

Patients with anti‐MDA5 + CADM‐ILD will have a higher mortality rate, and the initial 3 months after diagnosis of RPILD is considered the risk window for the dismal prognosis. Patients with CRP ≥ 10 mg/L, Ro52 (+) and RPILD may be related to a shorter survival time, while patients complicated with arthritis may present with relatively mild conditions.

## 1 INTRODUCTION

Clinically amyopathic dermatomyositis (CADM) is a distinct subtype of idiopathic inflammatory myopathy (IIM) characterized by signature skin lesions of dermatomyositis (DM), with no or only mild muscle involvement. Some CADM patients may progress to classical dermatomyositis (CDM).^1^ The prognosis of CADM heavily relies on complications, particularly interstitial lung disease (ILD). Certain patients may develop into the rapidly progressive interstitial lung disease (RPILD), which is resistant to glucocorticoids and immunosuppressants, and presents a high mortality rate.^2^ Melanoma differentiation‐associated gene 5 (MDA5) serves as a myositis‐specific antibody (MSA). Studies have demonstrated a higher positive rate of anti‐MDA5 antibody in CADM patients compared with CDM,^3^ which is closely associated with RPILD.^4^ The management of anti‐MDA5 + CADM combined with RPILD remains challenging in clinical practice.^5^ Despite significant advances in clinical management, intensified immunosuppressive therapy may be overdone for a considerable proportion of patients who may only present as chronic or stable cases.^6^ It is necessary to subdivide anti‐MDA5 + CADM‐ILD patients to identify those with dismal prognosis. Although similar to previous findings on anti‐MDA5 + DM, our findings are more applicable to patients with anti‐MDA5 + CADM‐ILD. In view of extremely rare research on anti‐MDA5 + CADM‐ILD at the current stage, this study aims to analyze the prognosis‐related risk factors among anti‐MDA5 + CADM‐ILD patients, with an effort to enhance understanding and diagnosis of the disease, and correspondingly reduce the mortality risk.

## 2 MATERIALS AND METHODS

### 2.1 Research subjects

The data set for this study included 125 patients with anti‐MDA5 + CADM‐ILD from the Nanjing Medical University myositis‐associated interstitial lung disease cohort (NMMI), recruited from December 2014 to August 2023 (the median duration of follow‐up was 12 months). NMMI is a multicenter, retrospective, and longitudinal cohort composed of patients from 10 tertiary hospitals in eastern China. Our study only analyzed patients with anti‐MDA5+ clinically amyopathic dermatomyositis combined with ILD and exclude those with classical dermatomyositis. The above patients all meet the 2017 EULAR/ACR classification criteria for inflammatory myopathy.^7^ The median age was 52 years, with the age division being based on the Chinese elderly standard of 60 years old.

### 2.2 Research contents

In this cohort study, patients were screened for MSAs and myositis‐associated antibodies (MAAs) using a commercial immunoblot (Euroimmun) assay with 18 autoantigens from the same central laboratory. Antinuclear antibody (ANA) were determined by the Nova Lite Hep‐2 ANA kit (Inova Diagnostics). All patients' medical records were reviewed retrospectively. ILD was diagnosed according to respiratory symptoms (dry cough and dyspnoea on exertion), physical examinations (such as Velcro rales in the lung bases) and high‐resolution computed tomography (HRCT) findings (substantial ILD findings such as ground‐glass attenuations, consolidations, reticulationsand/or honeycombing), with the exclusion of infection and drug‐induced interstitial changes. RPILD was defined based on the criteria proposed by Akira et al.,^8^ which included dyspnoea that worsened within 1 month, new lung opacities identified on chest HRCT, a decline in partial pressure of oxygen (PaO2) of >10 mmHg, and no evidence of infection, pulmonary embolism, congestive heart failure, or pneumothorax. Two expert thoracic radiologists independently reexamined all available HRCT images from baseline until the patient's death or last follow‐up visit. We collected baseline characteristics of patients, including demographic, clinical and laboratory data at the time of diagnosis. Laboratory indicators are grouped according to the upper limit of the normal range. Patient reassessments were conducted monthly for the first 3 months and then every 3‐6 months thereafter.

### 2.3 Statistical analysis

We conduct statistical analysis using R version 4.3.1. Kaplan‐Meier method was applied to calculate the cumulative survival rate and plot the survival curve. Univariate analysis was performed using the log‐rank test and chi‐square test, while multivariate analysis was conducted using logistic and cox analysis. *p* < 0.05 indicates statistically significant differences.

## 3 RESULTS

### 3.1 Mortality analysis of anti‐MDA5 + CADM‐ILD patients

The mortality rate among 125 anti‐MDA5 + CADM‐ILD patients in this cohort was 24.8% (31/125), with one patient lost to follow‐up. A total of 31 patients in our study cohort died, 26 died of respiratory failure, and 5 died of severe infection. During the follow‐up period, 47 patients (37.6%) experienced RPILD, among which 26 died during follow up, all within 6 months after diagnosis of RPILD. Among them, 73.1% (19/26) of deaths occurred within 1 month and 96.2% (25/26) within 3 months after diagnosis of RPILD. The mortality rates of anti‐MDA5 + CADM‐RPILD patients at 1 month, 2 months, and 3 months after diagnosis of RPILD were 40.4% (19/47), 51.1% (24/47), and 53.2% (25/47), respectively. In contrast, for those who survived for more than 3 months after the diagnosis of RPILD, the mortality rate was only 5.6% (1/18).

### 3.2 Risk analysis of RPILD in anti‐MDA5 + CADM‐ILD patients

Univariate analysis (chi‐square test) revealed that age ≥60 years, actinic rash, aspartate transaminase (AST) levels ≥40 u/L, creatine kinase (CK) levels ≥198 u/L, C‐reactive protein (CRP) levels ≥10 mg/L, serum ferritin (SF) levels ≥200 ng/mL or recombinant human tripartite motif containing 21 (Ro52) ( + ) increased the risk of developing RPILD. Other factors did not show statistical significance. Then we will incorporate statistically significant factors into the Logistic multivariate analysis. Logistic multivariate analysis showed that CRP levels ≥10 mg/L (*z* = 6.703, *p* = 0.010, *OR* = 5.853) and Ro52 (+) (*z* = 8.948, *p* = 0.003, *OR* = 8.951) were associated with a higher risk for developing RPILD. Other factors did not show statistical significance. The above was shown in Table 1.

**Table 1:** Univariate and multivariate analysis of the risk factors of rapidly progressive interstitial lung disease in anti‐MDA5 + CADM‐ILD patients.

Variable	Category	No‐RPILD	RPILD	Chi‐square test		Logistic multivariate analysis		
				*χ* ^2^	*P*	*OR*	*Z*	*P*
Gender	Male	21	15	0.356	0.551			
	Female	57	32					
Age (years)	<60	58	26	**4.824**	**0.028**	0.920	0.018	0.894
	≥60	20	21					
Gottron's sign	‐	27	17	0.031	0.860			
	+	51	30					
Actinic rash	‐	33	30	**5.434**	**0.020**	0.465	1.780	0.182
	+	45	17					
V‐sign	‐	49	29	0.016	0.900			
	+	29	18					
Shawl sign	‐	60	36	0.002	0.967			
	+	18	11					
Periungual erythema	‐	60	38	0.267	0.605			
	+	18	9					
Mechanic's hands	‐	56	32	0.194	0.660			
	+	22	15					
Arthritis	‐	40	31	2.574	0.109			
	+	38	16					
Superficial skin erosion and ulcer	‐	69	45	1.938	0.164			
	+	9	2					
ALT (u/L)	<40	35	19	0.373	0.541			
	≥40	41	28					
AST (u/L)	<40	35	12	**5.180**	**0.023**	2.060	1.275	0.259
	≥40	41	35					
LDH (u/L)	<245	22	8	2.240	0.134			
	≥245	54	39					
CK (u/L)	<198	66	10	1.000	0.317			
	≥198	36	9					
CRP (mg/L)	<10	64	24	**16.880**	**0.000**	**5.853**	**6.703**	**0.010**
	≥10	11	23					
SF (ng/mL)	<200 ng/mL	15	3	**5.553**	**0.018**	3.896	2.612	0.106
	≥200 ng/mL	38	34					
ESR (mm/h)	<20	19	4	**4.909**	**0.027**	1.886	0.633	0.426
	≥20	56	41					
MAD5	+ ~ ++	38	18	1.288	0.256			
	+++	40	29					
Ro52	‐	37	6	**15.622**	**0.000**	**8.951**	**8.948**	**0.003**
	+	41	41					
ANA	‐	38	17	1.874	0.171			
	+	40	30					
ARS	‐	74	42	1.333	0.248			
	+	4	5					

*Note*: Bold values indicate meaningful results.

Abbreviations: ALT, glutamic‐pyruvic transaminase; ANA, antinuclear antibody; ARS, anti‐synthetase antibody; AST, aspartate transaminase; CK, creatine kinase; CRP, C‐reactive protein; ESR, erythrocyte sedimentation rate; LDH, lactate dehydrogenase; MDA5, melanoma differentiation‐associated gene 5; Ro52, recombinant human tripartite motif containing 21; RPILD, rapidly progressive interstitial lung disease; SF, serum ferritin.

### 3.3 Risk analysis of survival time in anti‐MDA5 + CADM‐ILD patients

**Figure 1:**
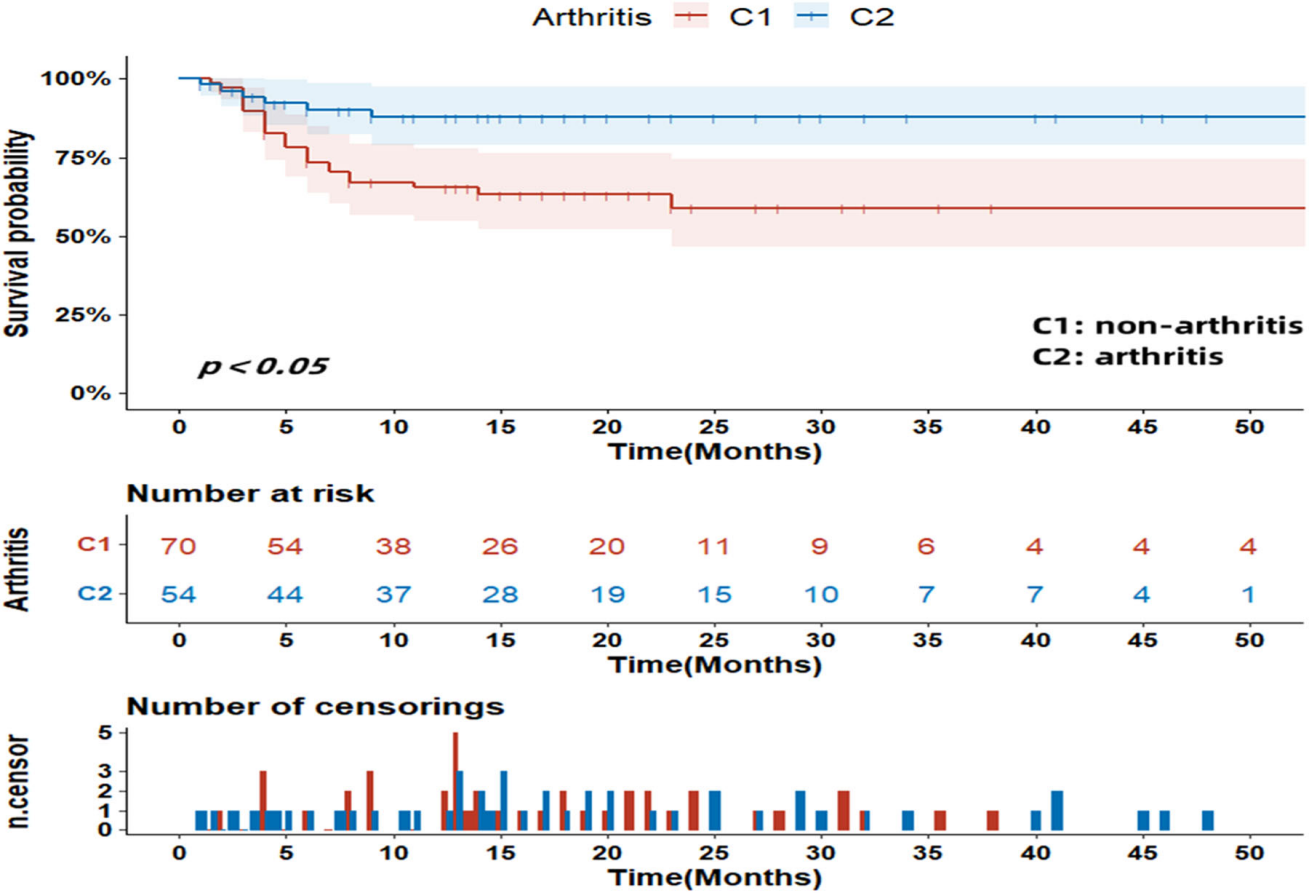
The influence of arthritis on survival time (months).

**Figure 2:**
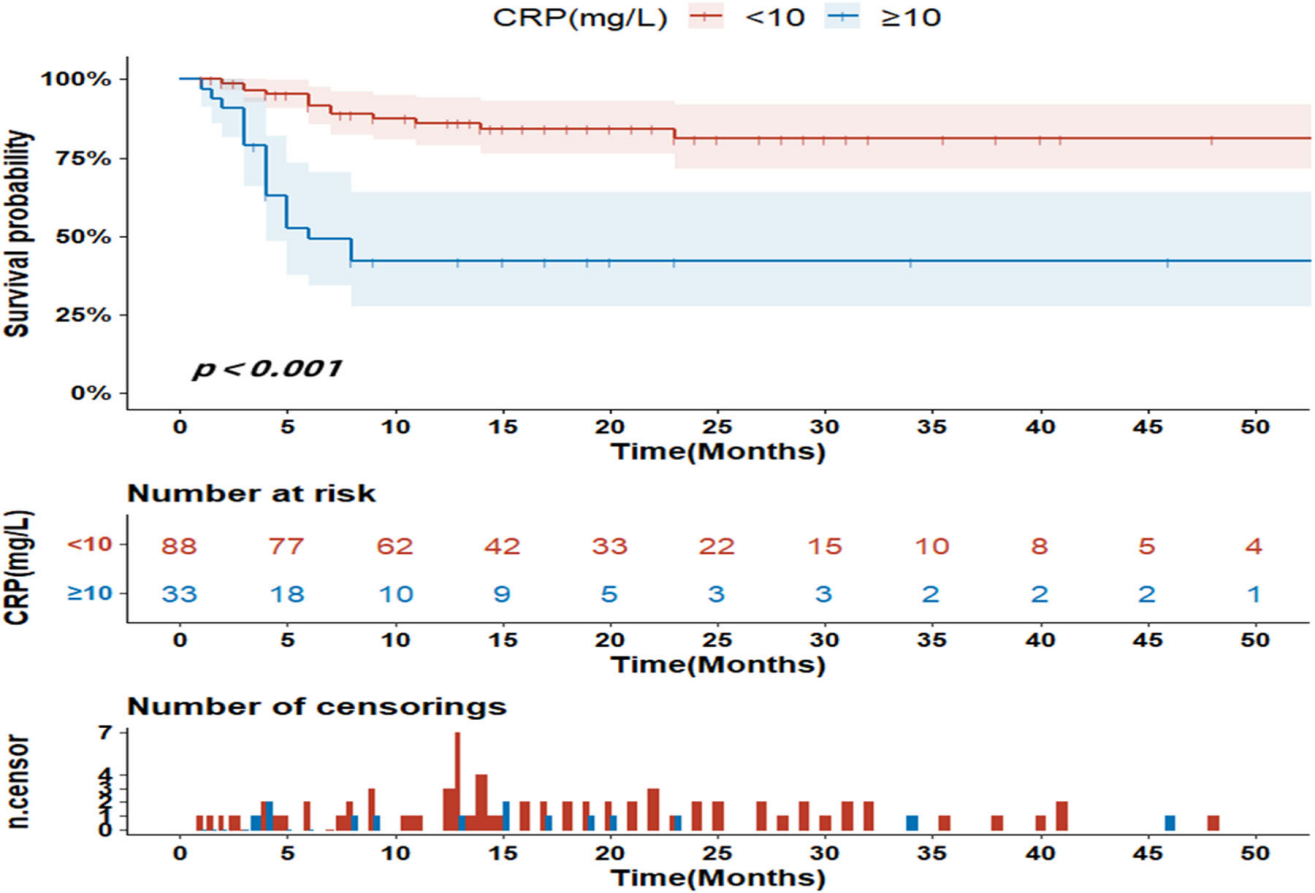
The influence of C‐reactive protein on survival time (months).

**Figure 3:**
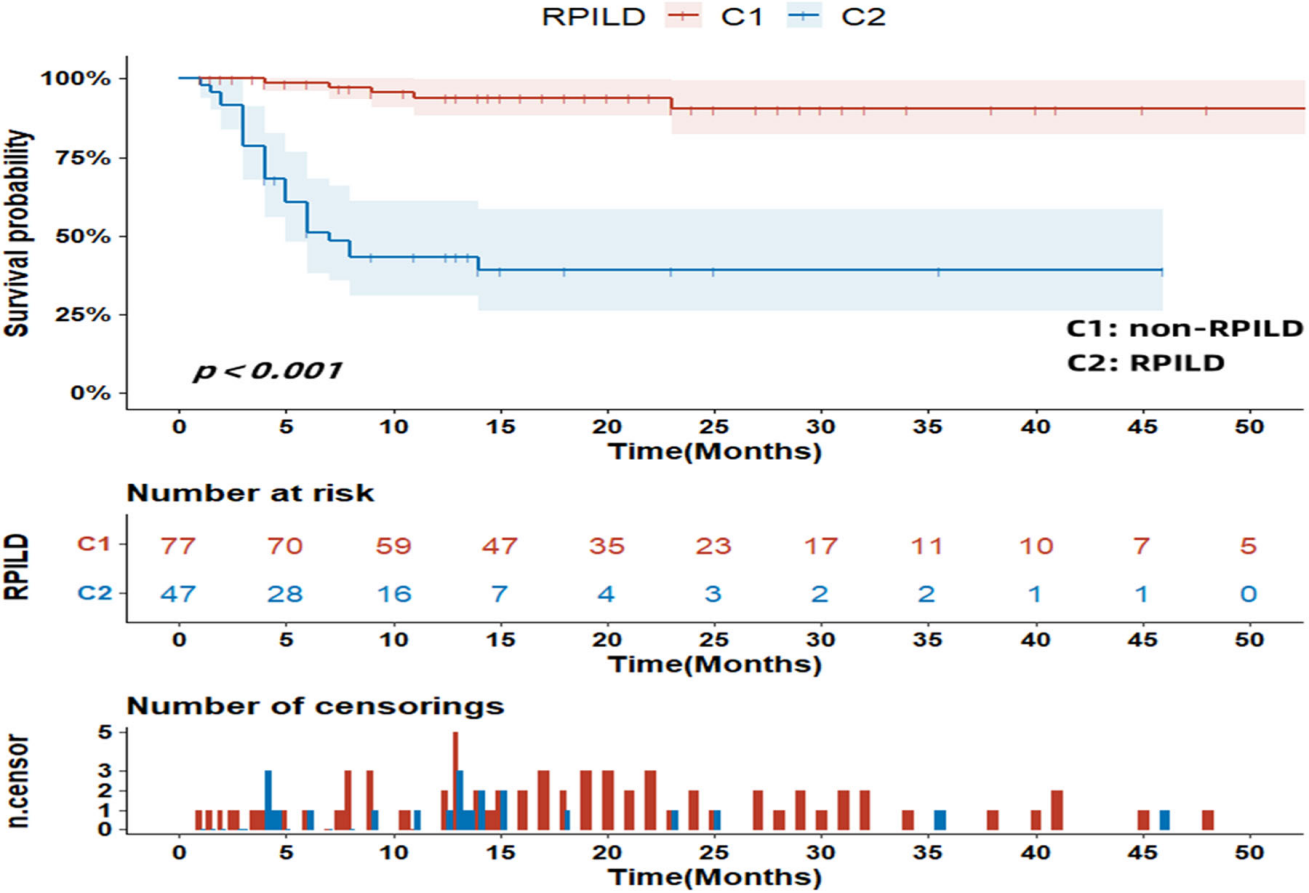
The influence of rapidly progressive interstitial lung disease on survival time (months).

Univariate analysis (log‐rank test) showed that: Age ≥60 years, AST ≥ 40 u/L, lactate dehydrogenase (LDH) ≥ 245 u/L, CK ≥ 198 u/L, CRP ≥ 10 mg/L, SF ≥ 60 years 200 ng/mL, high titers of MDA5 (+++), and RPILD might lead to shorter survival times for patients, whereas patients with arthritis had a lower risk of survival time (Figures 1, 2, 3). Other factors did not show statistical significance. Then we will incorporate statistically significant factors into the COX multivariate analysis. Cox multivariate analysis showed that: CRP levels ≥ 10 mg/L (*z* = 5.605, *p* = 0.018, *OR* = 3.547), RPILD (*z* = 8.878, *p* = 0.003, *OR* = 10.277) were associated with a shorter survival time, while arthritis (*z* = 5.754, *p* = 0.016, *OR* = 0.226) was independent protective factors. Other factors were not statistically significant. The above was shown in Table 2.

**Table 2:** Univariate and multivariate analysis of the influence factor of survival time in anti‐MDA5 + CADM‐ILD patients.

Variable	Category	No‐Death	Death	Log‐rank test		Cox multivariate analysis		
				*χ* ^2^	*P*	*OR*	*Z*	*P*
Gender	Male	25	11	0.835	0.361			
	female	68	20					
Age (years)	<60	70	13	**11.672**	**0.001**	1.239	0.201	0.654
	≥60	23	18					
Gottron's sign	‐	33	11	0.000	1.000			
	+	60	20					
Actinic rash	‐	43	19	2.108	0.147			
	+	50	12					
V‐sign	‐	54	23	2.570	0.109			
	+	39	8					
Shawl sign	‐	70	25	0.375	0.540			
	+	23	6					
Periungual erythema	‐	73	24	0.016	0.900			
	+	20	7					
Mechanic's hands	‐	68	21	0.116	0.734			
	+	27	10					
Arthritis	‐	45	25	**9.841**	**0.002**	**0.226**	**5.754**	**0.016**
	+	48	6					
Superficial skin erosion and ulcer	‐	85	28	0.033	0.855			
	+	8	3					
ALT (u/L)	<40	44	10	2.428	0.119			
	≥40	47	21					
AST (u/L)	<40	40	7	**4.461**	**0.035**	1.548	0.569	0.451
	≥40	51	24					
LDH (u/L)	<245	27	3	**4.984**	**0.026**	0.408	1.264	0.261
	≥245	64	28					
CK (u/L)	<198	81	21	**8.801**	**0.003**	1.361	0.287	0.592
	≥198	9	9					
CRP (mg/L)	<10	75	13	**19.923**	**0.000**	**3.547**	**5.605**	**0.018**
	≥10	15	18					
SF (ng/mL)	<200 ng/mL	17	1	**4.846**	**0.028**	2.122	0.477	0.490
	≥200 ng/mL	49	22					
ESR (mm/h)	<20	20	3	1.984	0.159			
	≥20	70	26					
MAD5	+ ~ ++	48	8	**6.252**	**0.012**	0.787	0.173	0.592
	+++	45	23					
Ro52	‐	36	7	2.670	0.102			
	+	57	24					
ANA	‐	42	12	0.394	0.530			
	+	51	19					
ARS	‐	85	30	0.998	0.318			
	+	8	1					
RPILD	‐	72	5	**37.107**	**0.000**	**10.277**	**8.878**	**0.003**
	+	21	26					
FVC %pred^a^	≥70%	45	14	1.605	0.205			
	<70%	9	6					
FEV_1_%pred^a^	≥80%	44	14	1.135	0.287			
	<80%	10	6					
FEV1/FVC^a^	≥70%	43	17	0.471	0.493			
	<70%	12	3					

*Note*: Bold values indicate meaningful results.

Abbreviations: ALT, glutamic‐pyruvic transaminase; ANA, antinuclear antibody; ARS, anti‐synthetase antibody; AST, aspartate transaminase; CK, creatine Kinase; CRP, C‐reactive protein; ESR, erythrocyte sedimentation rate; FEV1%pred, forced Expiratory Volume in the first second/predicted value ratio; FEV1/FVC, forced expiratory volume in one second/forced vital capacity; FVC %pred, fvcforced vital capcacity/predicted value ratio; LDH, lactate dehydrogenase; MDA5, melanoma differentiation‐associated gene 5; Ro52, recombinant human tripartite motif containing 21; RPILD, rapidly progressive interstitial lung disease; SF, serum ferritin.

^a^ Group by the lower limit of mild abnormalities.

### 3.4 Subgroup analysis based on the presence of arthritis and CRP levels

**Figure 4:**
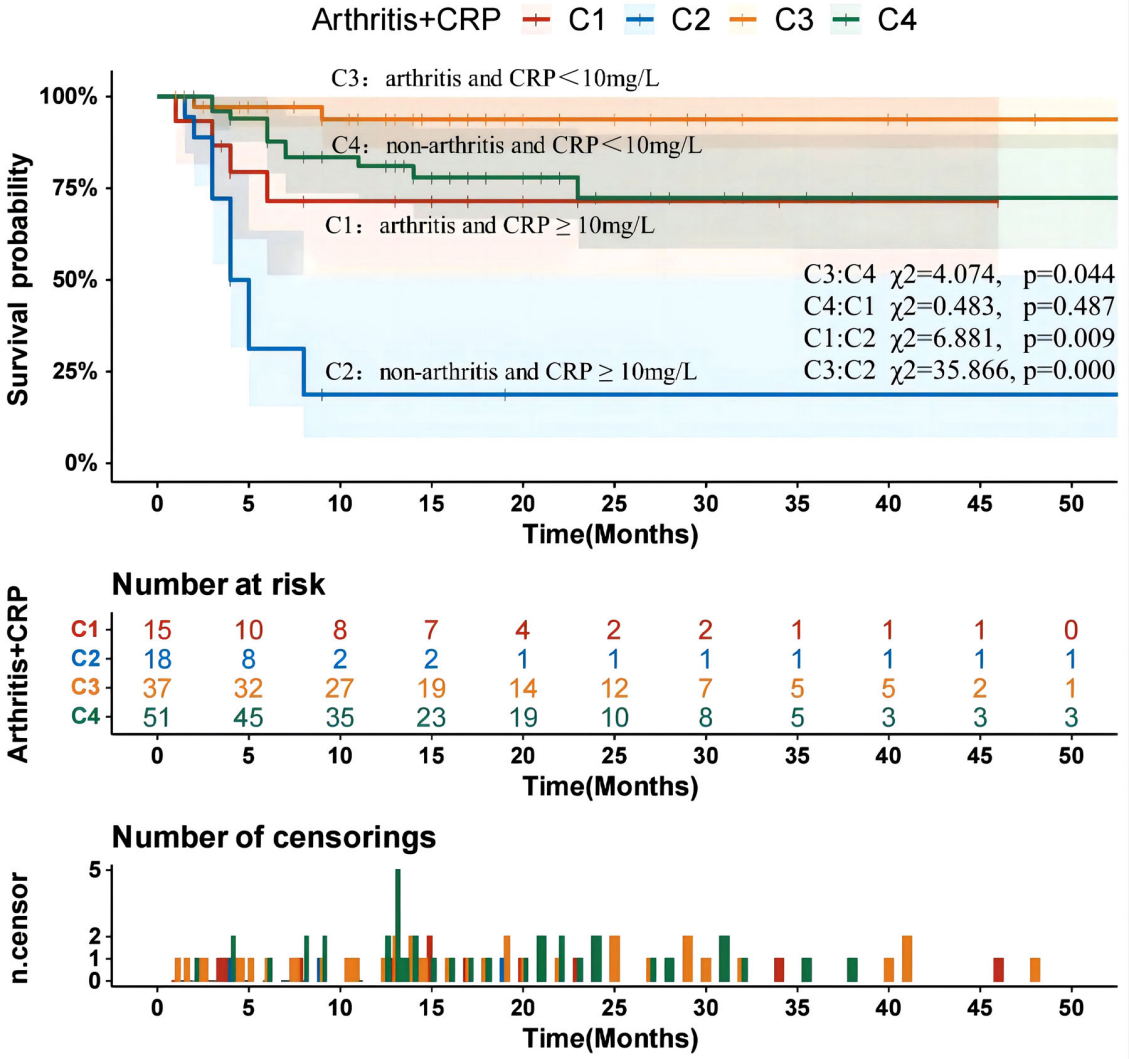
Survival analysis of subgroups based on the presence of arthritis and C‐reactive protein levels.

The results showed that patients with arthritis and lower CRP levels correlate with improved survival outcomes, compared to other anti‐MDA5 + CADM‐ILD patients. Patients without arthritis but with higher CRP levels have a worse prognosis. The above was shown in Figure 4.

## 4 DISCUSSION

Our retrospective cohort included a large number of anti‐MDA5 + CADM patients, with ILD as the main clinical manifestation, who were hospitalized in Rheumatology and Immunology Departments of 10 Class III hospitals in eastern China. Anti‐MDA5 + CADM is a clinical heterogeneous disease, with high morbidity of RPILD, short survival time, and sudden onset. Some patients may die of respiratory failure in a short period.^9^ There were currently some studies on the prognostic factors of anti‐MDA5 + DM, but research about anti‐MDA5 + CADM‐ILD was extremely rare. In the present study, retrospective analysis was carried out on the prognostic factors in 125 patients with anti‐MDA5 + CADM combined with ILD, and underlying clinical factors that could indicate dismal prognosis at diagnosis were identified, which has more precise clinical application value.

A meta‐analysis by Xie H et al.^10^ included 29 cohorts and a total of 2645 patients. The results indicated that elderly and male individuals were death‐related risk factors in anti‐MDA5 + DM/CADM patients. While our study suggested no value of gender and age in evaluating the prognosis of anti‐MDA5 + CADM‐ILD patients. Chen et al.^11^ found that anti‐MDA5 + CADM patients were more prone to fever, joint and skin symptoms, with the most common being periorbital rash and Gottron sign. Additionally, they were liable to respiratory complications. Lv et al.^12^ identified that active rash may serve as an independent protective factor for death. Anti‐MDA5 antibodies are significantly associated with the occurrence of skin ulcers, especially those located in the pulp of the fingers.^13^ Histopathological analysis have confirmed the existence of severe skin vascular lesions in anti‐MDA5 + DM/CADM patients.^14^ However, the significance of skin ulcers remains controversial in clinical practice. In the study by Cao et al.,^15^ Gottron sign was associated with a remarkable increase in the morbidity of RPILD and a decrease in survival in DM/CADM patients, while Nagashima et al.^16^ found no correlation between them in their cohort study. Our analysis targeting anti‐MDA5 + CADM patients have found that skin ulcers, rashes, periungular erythemata, and artisan hand were not associated with the incidence of RPILD and the survival rate of patients. Recently, numerous large‐scale studies have demonstrated the independent protective role of joint inflammation in the prognosis of anti‐MDA5 + DM patients.^17^, ^18^, ^19^ In our anti‐MDA5 + CADM cohort, 43.2% (54/125) of patients presented with arthritic symptoms, and RPILD exhibited a morbidity of 29.6%, lower than that of patients without joint symptoms (43.7%). The results suggested that arthritis may be an independent protective factor for anti‐MDA5 + CADM patients, which may be attributed to the fact that the inflammatory response in arthritis patients mainly occurs locally in the joints, with rare impact on other vital organs throughout the body.

In clinical practice, muscle enzyme profiles are typically used as an activity index of disease activity in CDM, but their value in CADM patients remains indefinite. Studies^10^, ^19^ demonstrated a lower survival rate in DM patients with CK ≥ 135 U/L. Zuo et al.^20^ suggested that elevated levels of glutamic‐pyruvic transaminase (ALT) and LDH were independent risk factors for RPILD in anti‐MDA5 + DM patients. Lian X et al.^21^ showed that LDH levels were associated with the progression of RPILD. In our study, no muscle enzyme was found to have statistical significance in predicting the prognosis of anti‐MDA5 + CADM patients. Although studies have shown that muscle enzymes are capable of predicting the prognosis of anti‐MDA5 + DM patients, their prognostic value remains questionable in CADM patients.

CRP is a nonspecific marker secreted by human liver organs during the acute systemic inflammatory response. Zou et al.^19^ revealed an independent correlation between elevated CRP and the occurrence of RPILD. Xie et al.^10^ and You et al.^22^ suggested that elevated CRP was a death‐related risk factor in anti‐MDA5 + DM patients. We have drawn a similar conclusion that higher CRP levels will further increase the risk of RPILD in anti‐MDA5 + CADM patients, along with an increased risk of mortality. For anti‐MDA5 + CADM‐ILD patients with CRP ≥ 10 mg/L, physicians should be highly vigilant about the risk of acute deterioration of their condition. No significant results were found between erythrocyte sedimentation rate (ESR) and prognosis, which may be attributed to its susceptibility to multiple factors.

SF, an acute‐phase protein secreted by macrophages, has been reported to serve as an evaluation indicator of disease activity in DM on the one hand, and a prognostic indicator for DM‐ILD on the other.^10^, ^20^, ^23^ Tanizawa et al.^24^ suggested that SF levels exceeding 500 ng/mL in DM‐RPILD patients indicated dismal prognosis. The study by Chen et al.^11^ demonstrated that anti‐MDA5 + CADM patients displayed higher levels of SF and ESR compared with those without anti‐MDA5, and anti‐MDA5 + CADM was more likely to trigger inflammatory responses, resulting in elevated SF levels. Moreover, SF levels will be particularly higher when complicated with ILD. However, our study on anti‐MDA5 + CADM‐ILD did not yield meaningful results. Nevertheless, patients with SF < 600 ng/mL, SF ≥ 600 ng/mL, SF < 1500 ng/mL and SF ≥ 1500 ng/mL presented a mortality of 7.3%, 40.8%, 15.7%, and 60.0%, respectively. It may be conducive to evaluating the prognosis of anti‐MDA5 + CADM‐ILD patients when improving the stratification criteria for SF.

Antibodies including anti‐MDA5 and anti‐Ro52 can serve as the serum antibody markers for patients with anti‐MDA5 + DM complicated with ILD to evaluate the prognosis.^25^, ^26^ You H et al.^27^ proposed that patients with high titers of anti‐MDA5 antibodies (++ ~  +++) exhibited an increased risk of RPILD, but no significant increase in the risk of mortality. While in our study, MDA5 antibody titer level was not an independent prognostic factor for patients with MDA5 + CADM. Therefore, we called in question its follow‐up value. Yamaguchi et al.^28^ demonstrated that anti‐MDA5 + CADM patients with other myositis‐related antibodies or other connective tissue diseases were less likely to develop RPILD and death compared to those with only positive anti‐MDA5 antibodies, suggesting that the presence of connective tissue diseases may serve as a protective factor for anti‐MDA5 + CADM‐ILD patients. In our cohort, 56.0% (70/125) of patients tested positive for ANA, and anti‐MDA5 + CADM‐RPILD patients showed a positive ANA rate of 63.8% (30/47). However, our study found no predictive value of both ANA and anti‐synthetase antibody (ARS) for prognosis. Furthermore, 54.5% (30/55) of ANA‐negative patients were positive for anti‐Ro52 antibodies, indicating that anti‐MDA5 antibody screening should be considered in ILD patients with negative ANA and positive anti‐Ro52.

Although anti‐Ro52 antibodies are initially identified as a serological marker for Sjogren's syndrome, it frequently coexists with myositis‐specific antibodies in idiopathic inflammatory myopathies rather than anti‐Ro60 antibodies.^29^ The clinical significance of anti‐Ro52 antibodies in myositis remains controversial. Patients with anti‐MDA5 + CADM‐ILD will have a worse prognosis under the condition of positive anti‐Ro52 antibodies compared with negative ones. Recently, Sabbagh et al.^30^ reported a significant increase in the frequency of anti‐Ro52 antibodies in adolescents with anti‐MDA5 + DM, which was associated with the presence of interstitial lung disease (ILD) and an unfavorable prognosis. The presence of anti‐Ro52 antibodies indicated a higher risk of severe conditions in patients with dermatomyositis (DM),^31^ suggesting that the positive anti‐Ro52 antibody is a more aggressive phenotype.^32^ Furthermore, Temmoku et al.^33^ suggested that DM patients with both positive anti‐MDA5 and anti‐Ro52 antibodies exhibited a higher mortality rate, emphasizing that caution should be exercised against such patients. Gan et al.^34^ proposed that the presence of anti‐Ro52 antibodies in CADM patients was linked to Raynaud's phenomenon and chronic ILD. Currently, there remains no consensus on whether the coexistence of anti‐MDA5 and anti‐Ro52 auto‐antibodies is associated with increased disease aggressiveness in CADM. In our cohort, patients with anti‐MDA5 + CADM‐ILD displayed a high prevalence (65.6%) of anti‐Ro52 antibodies, which increased the likelihood of RPILD and indicated a dismal prognosis in these individuals. The combination of anti‐MDA5 and anti‐Ro52 antibodies further stratified the survival outcomes of patients with anti‐MDA5 + CADM‐ILD, which may be conducive to enhancing the clinical management of such individuals.

Patients with anti‐MDA5 + CADM‐RPILD exhibited high mortality rates and unsatisfactory treatment outcomes.^35^ Clinically, physicians often correlate abnormal lung function data with a poor prognosis. In our study, some patients refuse to consent to undergoing lung function tests, and many critically ill patients could not have lung function tests due to their poor physical condition. The above may be the reason why we did not obtain meaningful results. If we incorporate patients who are unable to undergo lung function tests into the group of those with severe lung function abnormalities and then conduct statistical analysis, we may obtain meaningful results. ILD may be rapidly progressive in CADM patients. Matsuda et al.^36^ demonstrated that early intervention could significantly improve the survival rate for anti‐MDA5 + CADM/DM‐ILD patients, and the same was true for those implemented before the onset of ILD symptoms. In our study, RPILD showed a morbidity rate of 37.6%, and the majority of them presented with a rapid clinical progression. Among these cases after the the diagnosis of RPILD, 73.1% and 96.2% of deaths occurred within 1 month and 3 months, respectively. While patients who survived beyond 3 months showed a mortality rate of only 5.6%. It can be seen that the initial 3 months after diagnosis represent a critical risk window for nonideal prognosis in anti‐MDA5 + CADM‐RPILD patients. Those who survive beyond this relative risk period may embrace long‐term survival and are less likely to die due to disease recurrence, highlighting the importance of implementing initial treatment strategies for such patients.

Accurate risk assessment can significantly enhance the prognosis of high‐risk patients while avoiding overtreatment. Our research findings provide direct clinical implications: the initial 3 months following RPILD diagnosis are considered the risk period for death; CRP ≥ 10 mg/L and positive anti‐Ro52 antibodies may serve as independent risk factors for RPILD in anti‐MDA5 + CADM‐ILD patients; Patients with both CRP ≥ 10 mg/L and RPILD may exhibit an elevated mortality risk; Patients with arthritis may have a relatively favorable prognosis; Patients with arthritis and lower CRP levels correlate with improved survival outcomes. Although similar to previous findings on anti‐MDA5 + DM, our findings are more applicable to patients with anti‐MDA5 + CADM‐ILD. In this multi‐center study conducted in the Rheumatology and Immunology Departments of 10 tertiary class III hospitals in eastern China, a large numbers of samples were included, which provided unique advantages for the results. However, there are also certain limitations that need to be acknowledged, such as missing data for some patients, as well as deficient information related to lung‐function testing, lung biopsy, and treatment. Therefore, it is imperative to conduct population‐based multi‐center studies with larger scale and longer follow‐up duration, so as to obtain more comprehensive and accurate information. In conclusion, apart from typical skin symptoms, patients with anti‐MDA5 + CADM‐ILD also manifest pronounced pulmonary inflammatory responses. In view of this, early diagnosis, accurate evaluation, and timely treatment are indispensable to improve the prognosis of patients.

## AUTHOR CONTRIBUTIONS

Conceptualization and writing original draft: Wen Wang. Formal analysis and data curation: Wen Wang and Xiang Sun. Methodology: Xiang Sun. Supervision: Yan Xu and Wenfeng Tan. Writing review and editing: Ye Liu and Jun Zhou. All authors had full access to the data and had final responsibility for the decision to submit this manuscript for publication. All authors have read and approved the article. All authors have read and agreed to the published version of the manuscript.

## CONFLICT OF INTEREST STATEMENT

The authors declare no conflict of interest.

## ETHICS STATEMENT

This study was approved by the Ethics Committee of the First Affiliated Hospital of Nanjing Medical University (ethics approval information: 2020‐SR‐265) and complied with the terms of the Helsinki Declaration. Due to the retrospective nature of the study and the anonymity of the data, the requirement for informed consent was waived.

## Data Availability

The data that support the findings of this study are available upon request from the corresponding author. The data are not publicly available due to privacy or ethical restrictions.
